# Outcome and risk prediction of early progression in patients with extranodal natural killer/T cell lymphoma from the CLCG study

**DOI:** 10.1007/s00277-023-05311-5

**Published:** 2023-06-12

**Authors:** Jia-Ying Li, Xiao-Rong Hou, Si-Ye Chen, Xin Liu, Qiu-Zi Zhong, Li-Ting Qian, Xue-Ying Qiao, Hua Wang, Yuan Zhu, Jian-Zhong Cao, Jun-Xin Wu, Tao Wu, Su-Yu Zhu, Mei Shi, Hui-Lai Zhang, Xi-Mei Zhang, Hang Su, Yu-Qin Song, Jun Zhu, Yu-Jing Zhang, Hui-Qiang Huang, Ying Wang, Xia He, Li-Ling Zhang, Bao-Lin Qu, Yong Yang, Chen Hu, Min Deng, Shu-Lian Wang, Shu-Nan Qi, Ye-Xiong Li

**Affiliations:** 1 National Cancer Center/National Clinical Research Center for Cancer/Cancer Hospital, Chinese Academy of Medical Sciences and Peking Union Medical College, Beijing, 100021 China; 2 Peking Union Medical College Hospital, Chinese Academy of Medical Sciences (CAMS) and Peking Union Medical College (PUMC), Beijing, China; 3 Beijing Hospital, National Geriatric Medical Center, Beijing, China; 4 The Affiliated Provincial Hospital of Anhui Medical University, Hefei, Anhui China; 5 The Fourth Hospital of Hebei Medical University, Shijiazhuang, China; 6 Second Affiliated Hospital of Nanchang University, Nanchang, China; 7 Cancer Hospital of the University of Chinese Academy of Sciences (Zhejiang Cancer Hospital), Institute of Cancer and Basic Medicine (IBMC), Chinese Academy of Sciences, Zhejiang, China; 8 Shanxi Cancer Hospital and the Affiliated Cancer Hospital of Shanxi Medical University, Taiyuan, Shanxi China; 9 Fujian Provincial Cancer Hospital, Fuzhou, Fujian China; 10 Affiliated Hospital of Guizhou Medical University, Guizhou Cancer Hospital, Guiyang, Guizhou China; 11 Hunan Cancer Hospital and the Affiliated Cancer Hospital of Xiangya School of Medicine, Changsha, Hunan China; 12 Xijing Hospital of Fourth Military Medical University, Xi’an, China; 13 Tianjin Medical University Cancer Institute & Hospital, Key Laboratory of Cancer Prevention and Therapy, National Clinical Research Center for Cancer, Tianjin, China; 14 The Fifth Medical Center of PLA General Hospital, Beijing, China; 15 Key Laboratory of Carcinogenesis and Translational Research (Ministry of Education), Peking University Cancer Hospital & Institute, Beijing, China; 16 Sun Yat-sen University Cancer Center; State Key Laboratory of Oncology in South China; Collaborative Innovation Center for Cancer Medicine, Guangzhou, Guangdong China; 17 Chongqing University Cancer Hospital & Chongqing Cancer Hospital, Chongqing, China; 18 Jiangsu Cancer Hospital & Jiangsu Institute of Cancer Research, Nanjing, Jiangsu China; 19 Union Hospital, Tongji Medical College, Huazhong University of Science and Technology, Wuhan, Hubei China; 20 The General Hospital of Chinese People’s Liberation Army, Beijing, China; 21 Department of Radiation Oncology, Fujian Medical University Union Hospital, Fuzhou, Fujian China; 22 Division of Biostatistics and Bioinformatics, Sidney Kimmel Comprehensive Cancer Center, Johns Hopkins University School of Medicine, Baltimore, MD 21205-2013 USA

**Keywords:** NK/T cell lymphoma, Progression-free survival, Risk model

## Abstract

Recently, progression-free survival at 24 months (PFS24) was defined as clinically relevant for patients with extranodal NK/T cell lymphoma. Herein, the clinical data from two independent random cohorts (696 patients each in the primary and validation datasets) were used to develop and validate a risk index for PFS24 (PFS24-RI), and evaluate its ability to predict early progression. Patients achieving PFS24 had a 5-year overall survival (OS) of 95.8%, whereas OS was only 21.2% in those failing PFS24 (*P*<0.001). PFS24 was an important predictor of subsequent OS, independent of risk stratification. The proportion of patients achieving PFS24 and 5-year OS rates correlated linearly among risk-stratified groups. Based on multivariate analysis of the primary dataset, the PFS24-RI included five risk factors: stage II or III/IV, elevated lactate dehydrogenase, Eastern Cooperative Oncology Group score ≥2, primary tumor invasion, and extra-upper aerodigestive tract. PFS24-RI stratified the patients into low-risk (0), intermediate-risk (1–2), high-risk (≥3) groups with different prognoses. Harrell’s C-index of PFS24-RI for PFS24 prediction was 0.667 in the validation dataset, indicating a good discriminative ability. PFS24-RI calibration indicated that the actual observed and predicted probability of failing PFS24 agreed well. PFS24-RI provided the probability of achieving PFS24 at an individual patient level.

**Supplementary Information:**

The online version contains supplementary material available at 10.1007/s00277-023-05311-5.

## Introduction

Extranodal natural killer/T cell lymphoma (ENKTCL) is a rare, heterogeneous, and aggressive disease that shows a geographical and racial preference for East Asian and South American populations [1–3]. It predominantly originates in the upper aerodigestive tract (UADT), particularly the nasal cavity and Waldeyer’s ring [4, 5], and manifests as a localized disease with marked primary tumor invasion (PTI) [6–8].

Over the past decade, outcomes for patients with ENKTCL have improved because of the widespread application of upfront radiotherapy and modern chemotherapy regimens [9–16]. Five-year overall survival (OS) is 60–90% for localized disease and 20–40% for disseminated disease. However, about 10–30% of patients with early-stage disease and >50% of patients with advanced-stage disease will experience disease progression or relapse after treatment, with extremely poor survival outcomes [17–19]. Approximately 80% of disease progression or death occurs within 24 months after initial treatment [19]. The annual hazard rates of death and failure were highest in the first 24 months, but decreased to <5% from 36 months onward [19, 20]. Thus, we urgently require the tools for the early identification of progression in patients with ENKTCL. PFS at 24 months (PFS24) represents a logical cutoff time point to further evaluate treatment outcome and risk prediction for patients with ENKTCL.

Recently, we demonstrated that PFS24 is an early endpoint to evaluate disease-related outcomes in patients with ENKTCL [19]. Those who achieve PFS24 have a similar survival rate to the sex- and age-matched general population. By contrast, patients who have an early disease progression within the first 24 months have very poor outcomes [19]. Therefore, PFS24 is a promising endpoint to develop a risk model in patients with ENKTCL. In contrast to previously established prognostic models, such as the nomogram-revised risk index (NRI) and prognostic index of natural killer lymphoma (PINK), which use time-to-event endpoints [8, 21, 22], the dichotomous nature of the PFS24 endpoint permits the prediction of individual risk, and allows better modeling of the risk of disease related outcomes in a setting in which most patients are cured by first-line current treatment [23].

This present study aimed to determine the effect of PFS24 on OS. We developed a visual nomogram for individualized risk estimate of PFS24, constructed a personalized risk model for the endpoint PFS24, and carried out independent validation of its performance.

## Subjects and methods

### The study population and eligibility

From the China Lymphoma Collaborative Group (CLCG) database, we retrospectively reviewed patients with newly diagnosed ENKTCL from 2008 to 2016. Patients enrolled in this study received modern treatment strategies comprising non-anthracycline-based chemotherapy regimens and/or radiotherapy [14–16, 24]. Exclusion criteria for the study consisted of patients who received palliative treatment, anthracycline-based chemotherapy, or unknown chemotherapy regimens. A total of 1392 patients were included in this study. The study received ethical approval from the institutional review boards. The patient data were de-identified, precluding the requirement for informed patient consent.

Of 1315 patients with the primary site of UADT, most patients received radiotherapy with (*n* = 931; 70.8%) or without (*n* = 247; 18.8%) chemotherapy, whereas 137 (10.4%) patients received chemotherapy alone.

### Dataset grouping

The Ann Arbor staging system was used to stage the patients, and NRI and PINK were used for stratification [21, 22]. We evaluated the effect of PFS24 status on subsequent OS in all patients. To develop a risk index for PFS24 (PFS24-RI) and validate it independently, patients were randomly assigned to the primary dataset (*n* = 696) or the validation dataset (*n* = 696) in a 1:1 ratio using a stratified random sampling method.

### Statistical methods

PFS represented the time between treatment and initial disease progression, relapse, or any cause death. PFS24 meant that at 24 months after initial treatment, the patient was alive and had shown no disease progression or relapse. OS was defined as the time from treatment to any death from any cause. Subsequent OS after the risk-defining event was defined as the time from achieving PFS24 or time since progression in those patients who failed to achieve PFS24 to death from any cause. The Kaplan–Meier method was used to estimate the survival rates, which were compared using a log-rank test.

In the primary dataset, the independent clinical characteristics for PFS24 were determined using univariate and multivariate regression models. The validation dataset was used to assess the final model by measuring both discrimination and calibration. Model discrimination was evaluated using receiver operating characteristic curves and Harrell’s C-index. The integrated Brier score (IBS, a measure of the forecasting accuracy for probabilistic predictions) and a calibration curve were used determine the consistency between the nomogram-predicted survival probability and the actual observed outcome. Regression analysis was used to assess whether a linear relationship existed between PFS24 and OS. The Pearson correlation coefficient, *r*, was used to estimate the correlation in the weighted linear regression, which was weighted according to the number of patients. Analyses were conducted using IBM SPSS Statistics, version 27.0 (IBM Corp., Armonk, NY, USA, and the rms, hmisc, and survival packages in the *R* software version 4.0.5 (http://www.r-project.org). *P* < 0.05 (two-tailed) represented a statistically significant difference.

## Results

### Characteristics of the patients and their survival

Table 1 summarizes the patients’ baseline clinical characteristics. Their median age was 43 years old; and the ratio of males to females was 2.25:1. Most patients had a good performance status (PS) according to the Eastern Cooperative Oncology Group (ECOG) score 0–1 (95.2%), primary disease in the UADT (94.5%), and early-stage disease (90.2%). Lactate dehydrogenase (LDH) was elevated in 25.4% of the patients. Most of the patients were classified as low to intermediate-high risk based on NRI (85.0%) or low risk based on PINK (80.3%).

**Table 1:** Patient baseline characteristics according to achieving or failing PFS24

Characteristics	Achieved PFS24 (*n* = 1023)	Failed PFS24 (*n* = 369)	Total (*n* = 1392)
Sex			
Male	712 (69.6)	252 (68.3)	964 (69.3)
Female	311 (30.4)	117 (31.7)	428 (30.7)
Age (years)			
≤ 60	890 (87.0)	320 (86.7)	1210 (86.9)
> 60	133 (13.0)	49 (13.3)	182 (13.1)
Primary site			
UADT	989 (96.7)	326 (88.3)	1315 (94.5)
Extra-UADT	34 (3.3)	43 (11.7)	77 (5.5)
Regional lymph nodes			
No	687 (67.2)	201 (54.5)	888 (63.8)
Yes	336 (32.8)	168 (45.5)	504 (36.2)
Distant lymph nodes			
No	993 (97.1)	335 (90.8)	1328 (95.4)
Yes	30 (2.9)	34 (9.2)	64 (4.6)
Primary tumor invasion			
No	491 (48.0)	136 (36.9)	627 (45.0)
Yes	532 (52.0)	233 (63.1)	765 (55.0)
B symptoms			
No	645 (63.0)	218 (59.1)	863 (62.0)
Yes	378 (37.0)	151 (40.9)	529 (38.0)
Elevated LDH			
No	795 (77.7)	243 (65.9)	1038 (74.6)
Yes	228 (22.3)	126 (34.1)	354 (25.4)
ECOG score			
0–1	988 (96.6)	337 (91.3)	1325 (95.2)
≥ 2	35 (3.4)	32 (8.7)	67 (4.8)
Ann Arbor stage			
I	661 (64.6)	166 (45.0)	827 (59.4)
II	300 (29.3)	128 (34.7)	428 (30.7)
III–IV	62 (6.1)	75 (20.3)	137 (9.8)
NRI			
Low risk	287 (28.1)	48 (13.0)	335 (24.1)
Intermediate-low risk	320 (31.3)	101 (27.4)	421 (30.2)
Intermediate-high risk	262 (25.6)	100 (27.1)	362 (26.0)
High risk	112 (10.9)	75 (20.3)	187 (13.4)
Very high risk	42 (4.1)	45 (12.2)	87 (6.3)
PINK			
Low risk	821 (80.3)	245 (66.4)	1066 (76.6)
Intermediate risk	158 (15.4)	64 (17.3)	222 (15.9)
High risk	44 (4.3)	60 (16.3)	104 (7.5)

*PFS24* progression-free survival at 24 months, *UADT* upper aerodigestive tract, *LDH* lactate dehydrogenase, *ECOG* Eastern Cooperative Oncology Group, *NRI* nomogram-revised risk index, *PINK* prognostic index of natural killer lymphoma

With a median follow-up of 53 months, 369 of 1392 patients (26.5%) developed early progression within 24 months (failing PFS24), whereas 1023 patients (73.5%) showed no progression (achieving PFS24); 295 patients (21.2%) died. In all patients, the 5-year PFS and OS rates were 66.9% and 77.7%; in early-stage patients, they were 70.2% and 80.3%; and in advanced-stage patients, they were 35.6% and 50.9%, respectively.

### Effect of PFS24 status on subsequent OS

**Fig. 1:**
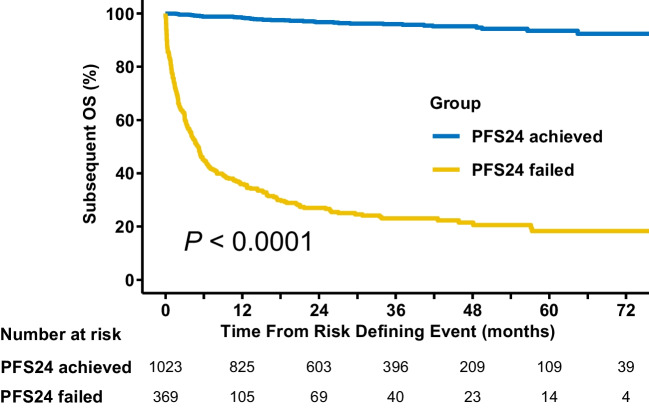
For all patients, the influence of PFS24 status on OS. The data show the OS of patients who failed or achieved PFS24. OS, overall survival; PFS24, progression-free survival at 24 months

We first assessed the impact of PFS24 status on long-term survival in all patients. The subsequent 5-year OS rate was 95.8% (95% confidence interval (CI): 94.3–97.3) for patients achieving PFS24, which was significantly higher than the 21.2% (95% CI: 16.8–26.9; *P* < 0.001) for those failing PFS24 (Fig. 1). After disease progression within the first 24 months, the median OS was only 5.1 (95% CI: 4.1–6.2) months.

### PFS24 as a predictor for subsequent OS independent of risk stratification

**Fig. 2:**
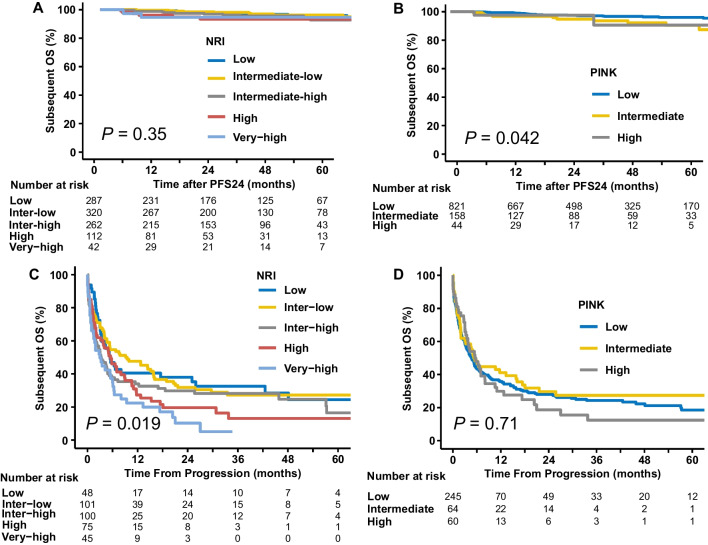
PFS24 as a predictor for subsequent OS, independent of risk-stratified groups, using prognostic models. Subsequent OS for patients achieving PFS24 in risk groups stratified by NRI (**A**) and PINK (**B**) models, and for patients failing PFS24 in risk groups stratified by NRI (**C**) and PINK (**D**) models. PFS24, progression-free survival at 24 months; OS, overall survival; NRI, nomogram-revised risk index; PINK, prognostic index of natural killer lymphoma

The impact of PFS24 status on subsequent OS in different risk groups was evaluated (Fig. 2). For patients achieving PFS24, the subsequent 5-year OS rate ranged from 92.8 to 94.7% across all risk groups stratified by NRI (Fig. 2A), and from 87.4 to 94.8% for all risk groups stratified by PINK (Fig. 2B). In contrast, for patients failing PFS24, the median OS was only 3 to 9.5 months for NRI-defined risk groups (Fig. 2C), and 4.7 to 5.5 months for PINK-defined risk groups (Fig. 2D). Patients achieving PFS24 had a very favorable prognosis, whereas patients failing PFS24 had a poor prognosis, regardless of the risk groups stratified using the NRI or PINK models.

### Correlation between the proportion of patients achieving PFS24 and OS in the risk-stratified groups

**Fig. 3:**
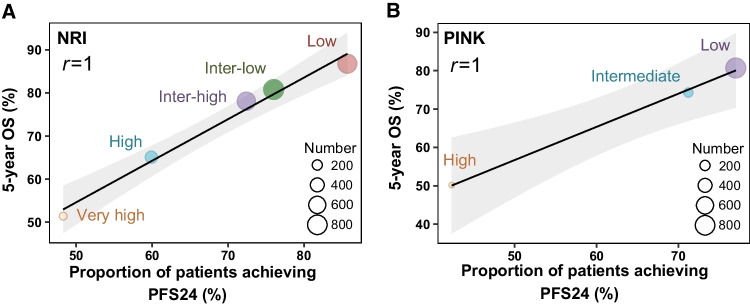
Correlation between the proportion of patients achieving PFS24 and the 5-year OS rate in different risk groups stratified by the NRI (**A**) and PINK (**B**) models. PFS24, progression-free survival at 24 months; OS, overall survival; NRI, nomogram-revised risk index; PINK, prognostic index of natural killer lymphoma

The proportion of patients who achieved PFS24 were 85.7%, 76.0%, 72.4%, 59.9%, and 48.3% in low-, intermediate-low-, intermediate-high-, high-, and very-high-risk groups stratified using NRI, respectively, with the corresponding 5-year OS rates of 86.8%, 80.8%, 78.1%, 65.1%, and 51.4%. Similarly, the proportion of patients achieving PFS24 were 77.0%, 71.2%, and 42.3% for low-, intermediate-, and high-risk groups stratified using PINK, respectively, with corresponding 5-year OS rates of 80.7%, 74.3%, and 50.2%, respectively. A significant linear correlation between the proportion of patients achieving PFS24 and 5-year OS rates was observed among different NRI-defined or PINK-defined risk groups (both *r* = 1, Fig. 3). These findings indicated that achieving PFS24 after first-line therapy is an important goal to obtain favorable long-term survival for an individual patient.

### Model development for PFS24-RI

We then developed a personalized risk model for PFS24. In the univariate analysis of the primary cohort, the risk factors associated with increased risk of disease progression before 24 months included extra-UADT (HR 3.34; 95% CI 1.78–6.32), regional lymph node involvement (HR 1.73; 95% CI 1.22–2.44), distant lymph node involvement (HR 3.55; 95% CI 1.82–7.02), presence of PTI (HR 1.67; 95% CI 1.18–2.37), elevated LDH (HR 1.99; 95% CI 1.37–2.89), ECOG score ≥2 (HR 2.95; 95% CI 1.44–6.07), and stage II (HR 1.70; 95% CI 1.15–2.49) or III–IV disease (HR 5.04; 95% CI 3.04–8.41; Supplemental Table 1). Multivariate analyses revealed that the stage, PTI, primary site, LDH, and ECOG score were independent risk factors for PFS24 (Table 2).

**Table 2:** Multivariable analysis of hazard ratios to achieve PFS24 in the primary dataset

Model definition	Variable	*P*	HR	95% CI	Nomogram score	Point
PFS24-RI	Primary site (Extra-UADT *vs*. UADT)	<0.001	1.99	1.62–6.43	58	1
Low risk (0)	PTI (yes *vs*. no)	0.022	1.41	0.97–2.08	29	1
Intermediate risk (1–2)	Elevated LDH (yes *vs*. no)	0.029	1.50	1.00–2.23	34	1
High risk (≥ 3)	ECOG score (≥ 2 *vs*. 0–1)	0.038	1.94	0.91–4.14	55	1
	Ann Arbor stage	<0.001				
	II (II *vs*. I)		1.47	0.97–2.20	32	1
	III/IV (III/IV *vs.* I)		3.31	1.89–4.12	100	2

*PFS24* progression-free survival at 24 months, *RI* risk index, *HR* hazard ratio, *CI* confidence interval, *UADT* upper aerodigestive tract, *PTI* primary tumor invasion, *LDH* lactate dehydrogenase, *ECOG* Eastern Cooperative Oncology Group

**Fig. 4:**
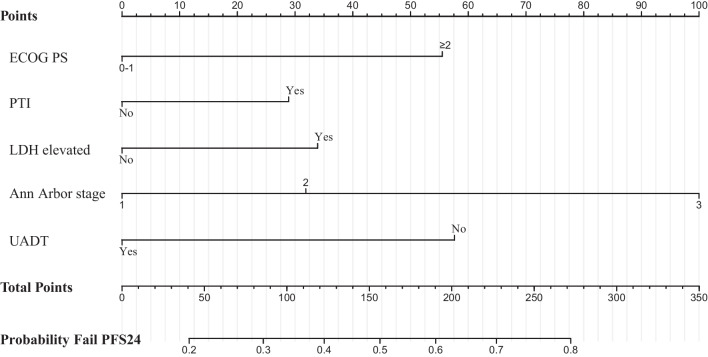
Nomogram predicting the probability of failing to reach PFS24 for patients with ENKTCL. Each covariable value corresponds to a "Points" score shown in the top line of the nomogram. The sum of the points, which is identified in the line "Total Points" shows the sum of the points, which is translated to predict the probability of failing PFS24. PFS24, progression-free survival at 24 months; ENKTCL, extranodal NK/T-cell lymphoma; ECOG, Eastern Cooperative Oncology Group; PS, performance status; LDH, lactate dehydrogenase; PTI, primary tumor invasion; UADT, upper aerodigestive tract

The multivariate analysis results were then used to develop an easy and visual prognostic nomogram to predict PFS24 (Fig. 4). Based on the corresponding regression coefficients, we then developed a PFS24-RI for the PFS24 endpoint in the primary dataset. The hazard ratio (HR) of the risk factors for PFS24 ranged between 1.4 and 2, with the exception of stage III/IV (HR = 3.31; Table 2). Consequently, we weighted the PFS24-RI components as follows: 1 point each for the risk factors extra-UADT; ECOG score ≥2; elevated LDH, PTI, or stage II; and 2 points for stage III/IV disease. Patients were stratified into the three groups based on the summed risk factor score: low risk (0), intermediate risk (1–2), and high risk (≥3).

### Survival outcomes in risk groups stratified by PFS24-RI

In the whole population in the primary dataset, stratification using the PFS24-RI categories classified 195 (28.0%) patients as low risk, 371 (53.3%) as intermediate risk, and 130 (18.7%) as high risk. In the validation dataset, PFS24-RI classified 188 (27.0%) patients as low risk, 387 (55.6%) as intermediate risk, and 121 (17.4%) as high risk. PFS24-RI identified a small proportion of patients at low-risk, whereas it identified that a large proportion of them were at intermediate or high risk of disease progression and death.

**Fig. 5:**
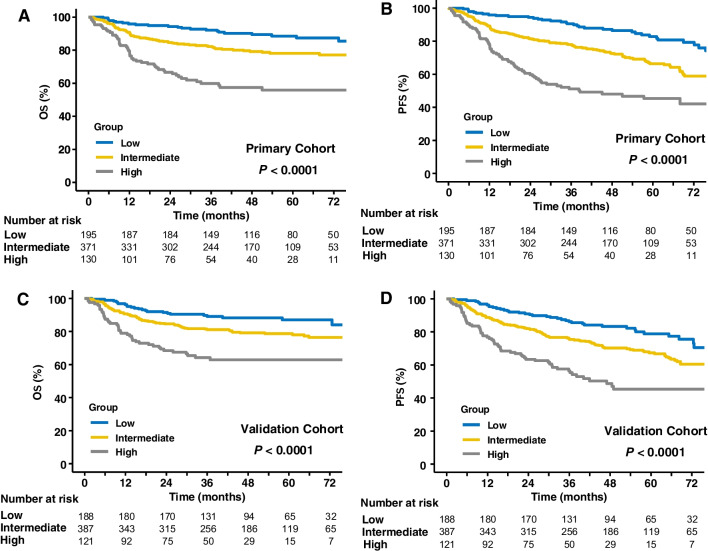
Survival curves of different risk groups based on PFS24-RI. OS (**A**) and PFS (**B**) of high, intermediate, and low-risk groups stratified according to PFS24-RI in the primary cohort. OS (**C**) and PFS (**D**) of high, intermediate, and low-risk groups stratified according to PFS24-RI in the validation dataset. PFS24, progression-free survival at 24 months; PFS24-RI, risk-index for progression-free survival at 24 months; OS, overall survival; PFS, progression-free survival

**Fig. 6:**
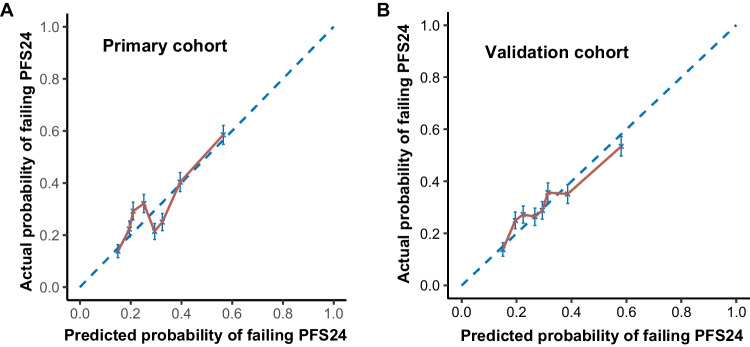
Calibration curves to predict the probability of achieving PFS24 in the primary (**A**) and validation (**B**) datasets. The *x*-axis shows the plotted predicted probability; the *y*-axis shows the plotted actual probability PFS24, progression-free survival at 24 months

Based on the PFS24-RI, the 5-year OS and PFS rates in three risk categories (low, intermediate, and high) were 87.7% (95% CI: 84.2–91.4) and 82.8% (95% CI: 87.0–89.0), 78.4% (95% CI: 75.4–81.6) and 66.4% (95% CI: 61.1–72.1), and 59.1% (95% CI: 52.8–66.1) and 45.3% (95% CI: 36.955.5) in the primary dataset (*P* < 0.001; Fig. 5A, B). The results were verified in the validation dataset, with 5-year OS and PFS rates of 87.0% (95% CI: 81.9–92.5) and 78.8% (95% CI: 72.3–85.9) in the low-risk group, 78.7% (95% CI: 74.5–83.1) and 67.3% (95% CI: 62.4–72.6) in the intermediate-risk group, and 62.9% (95% CI: 54.4–72.6) and 45.4% (95% CI: 36.3–56.7) in the high-risk group (*P* < 0.001; Fig. 6C, D). These results demonstrated that patients stratified by the PFS24-RI were associated with significantly different survival outcomes.

### Validation and predictive accuracy of PFS24-RI for OS

Using the validation dataset, computing the bootstrap C statistic and a calibration plot were used to independently validate PFS24-RI. The C-index of PFS24-RI for the prediction of PFS24 was 0.664 in the primary dataset and 0.667 in the validation dataset, indicating that PFS24-RI had a good discriminative ability. According to the calibration curve, PFS24-RI was well calibrated, with an IBS of 0.178 in the primary dataset and 0.185 in the validation dataset. For the probability of failing PFS24, the actual observed and PFS24-RI predicted results agreed well (Fig. 6).

## Discussion

Herein, we developed a PFS24-based risk model (PFS24-RI) to predict early disease progression in patients with ENKTCL under a current treatment strategy. A large multicenter cohort of patients was selected from the CLCG database to verify the PFS24 status as an indicator of prognosis of subsequent OS. Patients achieving PFS24 after initial therapy had favorable long-term outcomes, whereas patients failing PFS24 had poor prognosis, independent of risk groups stratified by the NRI or PINK models. Furthermore, extra-UADT, ECOG score ≥2, PTI, elevated LDH, and stage II or III–IV disease were identified as risk factors of failing PFS24, thus forming the PFS24-RI model. Based on PFS24-RI, patients could be categorized into low-, intermediate-, and high-risk groups with different prognoses. This model is useful to distinguish ENKTCL patients with different risks with disparate likelihoods of early progression or death. These results provide supplementary accuracy to the continual early estimation of prognosis after initial treatment, providing the rationale for surveillance strategies, and will help to guide prospective clinical trial designs for patients with ENKTCL.

In this study, using data from the CLCG database, we showed a similar frequency of early progression to that observed in previous studies [14, 17–19]. Among 1392 patients with ENKTCL, around a quarter (26.5%) experienced disease progression within 24 months after current treatment. PFS24 since initial therapy was associated with a significantly increased risk of death. There was a strong linear correlation between the proportion of patients achieving PFS24 and long-term OS among different risk groups stratified by NRI or PINK. Previously, we showed that prolonged OS is associated with improved PFS [25], and that survival time increased, the probability of survival increased, and the risks of failure and death decreased in early-stage ENKTCL [20]. Initially, the estimated risk of disease progression and mortality was high; however, they decreased markedly within the first 24 months after current treatment for ENKTCL [19, 20]. Patients who remained progression-free within 24 months after initial treatment had favorable long-term outcomes, with OS rates that were indistinguishable from those of age-, sex-, and country-matched populations [19]. In the present study, despite the heterogeneous prognoses for the NRI or PINK risk-stratified groups, all patients achieving PFS24 after initial treatment attained an equivalent favorable survival probability (~90%), independent of their initial risk category; however, patients failing PFS24 had extremely poor survival probability (median, ~6 months). Consistently, early progression within 24 months after initial therapy allowed stratification of subsequent OS and identified a high-risk population among patients with B cell lymphomas [26–30].

Optimization of risk stratification for early endpoints such as PFS24 and event-free survival at 24 months (EFS24) are crucial to facilitate prognosis and treatment decisions regarding lymphomas [27, 31, 32]. Here, we proposed that the novel PFS24-RI model, which integrated the established clinical risk factors to predict the PFS24 endpoint, will be useful in the upfront identification of high-risk patients with ENKTCL. The five variables, including stage, LDH, PS, primary site, and PTI, in the PFS24-RI are all well-established clinical predictors of survival outcome, and reflect the tumor load, invasive potential, and ability to tolerate treatment in patients with ENKTCL. LDH, stage, PS, and PTI remain from the NRI model [21], and primary site and stage are from the PINK model [22]. PFS24-RI provided a useful tool to advise patients for personalized risk prediction of early progression and has implications for enhancing patient stratification strategies in prospective clinical trial design. Patients at intermediate and high risk of disease progression within 24 months may benefit from escalated treatment or maintenance therapy.

Patient age is the most common indicator of continuous time-to-event outcomes, such as OS, in ENKTCL and other lymphomas [21, 33–35]. Consistently, elderly patients with ENKTCL had generally poor prognoses [36, 37]. However, older age (> 60 years old) was not identified as an independent risk factor in the PFS24-RI model. Our previous study demonstrated that elderly patients with low-risk early-stage disease or those with high-risk early-stage disease who achieve PFS24 after radiotherapy have similar survival to that of the sex- and age-matched general population [38]. These results suggested that, in contrast to the time-to-event endpoint of OS, PFS24 is less influenced by age and focuses more on disease events. Optimal treatment for ENKTCL should depend on disease-related risk factors rather than chronological age [37, 38]. Thus, PFS24-RI could generate predictions across a variety of ages as a disease-specific endpoint and could help to formulate more appropriate treatment strategies.

In this study, PFS24-RI clearly showed that patients with primary extra-UADT sites were at high risk of early progression. Previous studies demonstrated that a higher proportion of patients with extra-UADT ENKTCL had a risk of advanced-stage disease, elevated LDH, and poorer outcome compared with those with UADT ENKTCL [3, 4, 21, 22]. The reported 3-year OS and PFS rates were 43.6% and 27.9%, retrospectively [39]. This finding indicated that patients with extra-UADT ENKTCL were at increased risk of early progression and presented more aggressive clinical behavior.

This study included several strengths and limitations. Previous research highlighted more explicitly the importance of initial prognosis and treatment in patients with ENKTCL by using PINK and NRI at diagnosis [21, 22]. These previous models were constructed based on proportional hazards regression of time-to-event endpoints often, such as PFS or OS at 3 or 5 years, and assumed a constant risk of covariate implicitly. These models categorized patients into different risk groups according to outcomes provided by a survival curve; therefore, at specific time points, the predicted outcomes can vary markedly depending on clinical features of patients with ENKTCL in the original dataset. Thus, although a patient with ENKTCL can be classified as low risk or high risk, it is difficult to council patients on their individual risks for disease-specific outcomes. PFS24 has clinical relevance and could be adopted as a clinical study endpoint/early endpoint in a prognostic model of early progression prediction in ENKTCL. In this study, variables for potential inclusion in the PFS24-RI model were restricted to standard clinical variables that would be routinely available to physicians. This was a limitation of the model; however, the clinical variables included in the model have long history of clinical relevance in ENKTCL and are thus unlikely to be false associations. Although PFS24-RI is a personalized risk model to predict poor biological behavior, the precise mechanisms underlying early progression remain undefined. Assessment of tumor genetic features or including tumor biomarkers would be likely to add predictive information for patients with ENKTCL [40]. Further studies are needed to determine the effect of asparaginase-based regimens on PFS24 and OS, and assess the role of salvage treatments such as hematopoietic stem cell transplantation and immunotherapy for relapsed or refractory disease.

In conclusion, we demonstrated that PFS24 was an important predictor for OS, independent of NRI- or PINK-stratified risk groups. The PFS24-RI model provided the probability of achieving PFS24 at the level of the individual patient and could be applied for prospective study design and risk stratification.

## Supplementary information

ESM 1

## Data Availability

The data that support the findings of this study are available from the corresponding author upon reasonable request.
