# Supplementary material for: Outcome and risk prediction of early progression in patients with extranodal natural killer/T cell lymphoma from the CLCG study
Source: Ann Hematol. 2023 Jun 12;102(9):2459–69. doi: 10.1007/s00277-023-05311-5 (PMC10444649; doi:10.1007/s00277-023-05311-5)
Supplement: Supplementary file 1 — ESM 1 [file 277_2023_5311_MOESM1_ESM.docx]

**Supplemental Table 1.** Univariate logistic regression analyses for PFS24 in the primary dataset

|  | Patients |  | Failing PFS24 |  |  |  |
| --- | --- | --- | --- | --- | --- | --- |
| Variable | No. (%) |  | % |  | HR (95% CI) | *P* |
| Sex |  |  |  |  |  | 0.205 |
| Male | 498 (71.6) |  | 25.1 |  | Ref |  |
| Female | 198 (28.4) |  | 29.8 |  | 1.27 (0.88–1.82) |  |
| Age (years) |  |  |  |  |  | 0.654 |
| ≤ 60 | 608 (87.4) |  | 26.2 |  | Ref |  |
| > 60 | 88 (12.6) |  | 28.4 |  | 1.12 (0.67–1.82) |  |
| Primary site |  |  |  |  |  | < 0.001 |
| UADT | 654 (94.0) |  | 24.8 |  | Ref |  |
| Extra-UADT | 42 (6.0) |  | 52.4 |  | 3.34 (1.78–6.32) |  |
| Regional lymph nodes |  |  |  |  |  | < 0.001 |
| No | 448 (64.4) |  | 22.5 |  | Ref |  |
| Yes | 248 (35.6) |  | 33.5 |  | 1.73 (1.22–2.44) |  |
| Distant lymph nodes |  |  |  |  |  | < 0.001 |
| No | 659 (94.7) |  | 24.9 |  | Ref |  |
| Yes | 37 (5.3) |  | 54.1 |  | 3.55 (1.82–7.02) |  |
| Primary tumor invasion |  |  |  |  |  | < 0.001 |
| No | 309 (44.4) |  | 21.0 |  | Ref |  |
| Yes | 387 (55.6) |  | 30.7 |  | 1.67 (1.18–2.37) |  |
| B symptoms |  |  |  |  |  | 0.142 |
| No | 436 (62.6) |  | 24.5 |  | Ref |  |
| Yes | 260 (37.4) |  | 29.6 |  | 1.29 (0.92–1.82) |  |
| Elevated LDH |  |  |  |  |  | < 0.001 |
| No | 530 (76.1) |  | 23.0 |  | Ref |  |
| Yes | 166 (23.9) |  | 37.3 |  | 1.99 (1.37–2.89) |  |
| ECOG score |  |  |  |  |  | < 0.001 |
| 0–1 | 664 (95.4) |  | 25.3 |  | Ref |  |
| ≥ 2 | 32 (4.6) |  | 50.0 |  | 2.95 (1.44–6.07) |  |
| Ann Arbor stage |  |  |  |  |  | < 0.001 |
| I | 413 (59.3) |  | 19.6 |  | Ref |  |
| II | 205 (29.5) |  | 29.3 |  | 1.70 (1.15–2.49) |  |
| III–IV | 78 (11.2) |  | 55.1 |  | 5.04 (3.04–8.41) |  |
| NRI |  |  |  |  |  | < 0.001 |
| Low risk | 168 (24.1) |  | 13.7 |  | Ref |  |
| Intermediate-low risk | 210 (30.2) |  | 23.3 |  | 1.92 (1.13–3.35) |  |
| Intermediate-high risk | 181 (26.0) |  | 25.4 |  | 2.15 (1.25–3.79) |  |
| High risk | 93 (13.4) |  | 46.2 |  | 5.42 (3.01–10.01) |  |
| Very high risk | 44 (6.3) |  | 52.3 |  | 6.90 (3.33–14.62) |  |
| PINK |  |  |  |  |  | < 0.001 |
| Low risk | 527 (75.7) |  | 22.2 |  | Ref |  |
| Intermediate risk | 110 (15.8) |  | 30.0 |  | 1.50 (0.94–2.35) |  |
| High risk | 59 (8.5) |  | 57.6 |  | 4.77 (2.75–8.38) |  |

PFS24, progression-free survival at 24 months; UADT, upper aerodigestive tract; LDH, lactate dehydrogenase; ECOG, Eastern Cooperative Oncology Group; NRI, nomogram-revised risk index; PINK, prognostic index of natural killer lymphoma.
